# Illness Management & Recovery (IMR) in the Netherlands; a naturalistic pilot study to explore the feasibility of a randomized controlled trial

**DOI:** 10.1186/s12888-016-1096-y

**Published:** 2016-11-09

**Authors:** Bert-Jan Roosenschoon, Jaap van Weeghel, Moniek Bogaards, Mathijs L. Deen, Cornelis L. Mulder

**Affiliations:** 1 Department of Psychiatry, Epidemiological and Social Psychiatric Research Institute, Erasmus University Medical Centre, ‘s-Gravendijkwal 230, 3015 CE Rotterdam, The Netherlands; 2 Parnassia Psychiatric Institute, Parnassia Academy, Kiwistraat 32, 2552 DH Den Haag, The Netherlands; 3 Tilburg School of Social and Behavioral Sciences, Department of TRANZO, Tilburg University, Warandelaan 2, 5037 AB Tilburg, The Netherlands; 4 Parnassia Psychiatric Institute, Dijk en Duin, Oude Parklaan 125, 1901 ZZ Castricum, The Netherlands; 5 Parnassia Psychiatric Institute, Bavo-Europoort, Prins Constantijnweg 48-54, 3066 TA Rotterdam, The Netherlands; 6 Faculty of Social and Behavioral Sciences, Institute of Psychology, Leiden University, Wassenaarseweg 52, 2333 AK Leiden, The Netherlands

**Keywords:** Illness Management and Recovery, IMR, Self management, Severe mental Illness, Schizophrenia, Pilot study, Feasibility, Recovery

## Abstract

**Background:**

Illness Management & Recovery (IMR) is a curriculum-based program for people with severe and persistent mental illness. To date, four randomized controlled trials (RCTs) have been published on it. As these produced mixed results, we conducted a pilot study to test the feasibility of conducting a new RCT in a Dutch psychiatric institute. Because our primary objective was to evaluate support for implementing IMR on a broader scale, we examined participant recruitment, client outcomes, and clients’ and clinicians’ satisfaction. Secondary objectives were to evaluate fidelity, trainers’ training and supervision, and to explore program duration, dropout, and client characteristics related to dropout. For reporting, we used the checklist for pilot studies adopted from the CONSORT Statement.

**Methods:**

This program evaluation included a process-evaluation and an outcome evaluation with a One Group Pre-Posttest Design (*N* = 81). Interviews and internal reports were used to monitor participant numbers, program duration, dropout, and completers’ characteristics. Clients’ and clinicians’ satisfaction and provision of trainers’ training and supervision were assessed through interviews. Fidelity was assessed on the IMR Fidelity Scale; client outcomes were assessed on the IMR scale (client and clinician versions) and the Recovery Markers Questionnaire (RMQ).

**Results:**

Eighty-one participants were recruited of 167 people who were assessed for eligibility. Completers and clinicians were satisfied, and scores for completers improved significantly on the IMR scale (clinician version) (*d* = 0.84) and RMQ (*d* = 0.52), and not significantly on the IMR scale client version (*d* = 0.41). Mean fidelity was good, but three groups had only moderate fidelity. Our feasibility criterion for trainers’ education and supervision was partly attained. Dropout from treatment was 51 %; female participants and people who scored higher on both IMR-scales at baseline had a significantly lower chance of dropping out. The duration of IMR varied (*M* = 12.7 months, *SD* = 2.87).

**Conclusions:**

Results suggested that feasibility of conducting an RCT on IMR was good. Special attention is required to fidelity, IMR duration, trainers’ education and supervision, and dropout, especially of men. One study limitation was our inability to conduct follow-up measurements of non-completers.

## Background

### Introduction

The aim of Illness Management and Recovery (IMR) is to provide a structured psychosocial program that helps individuals to manage the disabling effects of severe and persistent mental illnesses such as schizophrenia and bipolar disorders. It is curriculum based. To improve different aspects of illness management, it includes interventions such as goal-setting, psycho-education, and coping and social skills training. The overall aim is to improve illness outcomes and to support subjective and objective recovery [1]. IMR is based on a review of controlled research on professionally based programs for helping people to manage their mental illness [2]. It was developed in the United States [3, 4] and is currently used in several countries. While its individual components are not new in Dutch Mental Health Care, what is new of this program is to offer these services together as an integrated package.

A review conducted in 2011 showed that three randomized controlled trials (RCTs), three quasi-controlled trials and three pre-post trials had been conducted on the overall IMR program [5]. To date (March 2016), four RCTs have been published on this program [6–9]. The results were mixed. The three RCTs that had been published before the start of our pilot [6–8] differed from each other with regard to setting, the number of participants and diagnoses, the length and format of IMR, the trainers’ training and their qualifications, the frequency of supervision of the trainers, the number and timing of measurements and the fidelity of implementation of IMR [10].

The same three RCTs compared IMR with care as usual (CAU) [6–8]. On the overall score of the client version of the Illness Management and Recovery Scale (IMRS) [11–13], two of them showed significantly positive results for clients assigned to IMR relative to those in the control groups; the respective effect sizes were .36 [7] and .29 [8]. The other study found a significant improvement only if the analyses were limited to sites with high IMR fidelity [6]. On the overall score of the clinician version of the IMRS [11–13], all three of these studies showed significantly positive results for clients assigned to IMR relative to those in the control groups, with respective effect sizes of .28 [6], .39 [7] and .34 [5, 8]. Additional significantly positive results for IMR were found on client-reported knowledge in one study [6], on client-reported coping in another study [8], on clinician-reported quality of life in the third study [7], and on observer-rated psychiatric symptoms in two of these studies [7, 8]. These results were either not found in the other RCTs, or the domains in question were not measured. No significant outcomes were found on objective outcomes such as medication dosage, employment, or number of hospitalizations [10]. The fourth, (i.e. later), RCT by Salyers et al. [9] compared IMR with an active control group. This measured psychiatric symptoms, quality of life, illness self-management, patient activation, medical adherence, perceived recovery, hope, and service utilization. No significant differences were found between the outcomes of IMR and those of a problem-solving group. Seventy-two percent of the participants did not complete the IMR program. Due to these mixed results, more research is needed on the outcomes of IMR.

In our main study we aim to study the effects of IMR in a Dutch context in a randomized multi-centre, single-blinded, clinical trial intended to compare IMR with treatment as usual for outpatient clients with severe and persistent mental illness. We will investigate whether IMR leads to better illness management, fewer symptoms and fewer relapses, and also to better subjective and objective recovery. Our study design was inspired by Mueser’s Conceptual Framework for IMR, which is claimed to affect many aspects of illness management and recovery [1, 10].

We expect the planned RCT to produce positive results on both of the IMR scales, similar to those found in the earlier studies that used CAU as a control [6–8] (see above). We also hope to gain additional information with regard to symptoms, coping and recovery, on which the earlier results differed.

The two main differences between our planned RCT and previous studies are 1.) that we will use various outcome measures on illness management, illness outcomes and recovery to provide a thorough measurement of the effects of IMR; and 2.) by testing our hypotheses, we will test various associations supposed by Mueser et al. [1] in their conceptual framework. By testing various components of the model, it might thus be said that we will test the model as a whole.

#### Rationale for assessing feasibility through piloting

Before deciding whether an RCT could be conducted, we evaluated the implementation of IMR in a pilot study intended to explore the feasibility of an RCT and to provide practical guidelines for the optimal implementation of IMR in a Dutch setting. Feasibility can be defined as “the extent to which a practice can be used or carried out within a setting” [14]. Feasibility studies are pieces of research done before a main study. They are used to estimate important parameters that are needed to design the main study [15]. Sometimes feasibility studies and pilot studies are not distinguished from each other [16].

We considered the support base for implementing IMR on a broader scale to be important to the feasibility of an RCT, as this would affect the number of participants who could be recruited for the RCT and because it would be executed in the same institute. This support base would be affected by participants’ and clinicians’ satisfaction with IMR and by its perceived effectiveness.

As the feasibility of such an RCT would also depend on the quality with which IMR was implemented, we chose to investigate aspects of implementation on which the earlier RCTs had differed, such as fidelity, trainers’ education and supervision, dropout from IMR, and IMR duration.

Our pilot study was designed as a program-evaluation that included a process evaluation and an outcome evaluation. As there have recently been calls for increased scientific rigor in pilot and feasibility studies [17], our reporting of this study is based on “Checklist: Items to include when reporting a pilot study” [16] adopted from the CONSORT format [18].

## Methods

### Participants and setting

In the pilot study we included adult outpatient clients aged between 18 and 65 with severe and persistent mental illnesses (SMI), i.e., those entailing serious limitations in social functioning and requiring coordinated professional care [19]. As well as behavior that would be likely to seriously disrupt the group, the exclusion criteria were having severe cognitive impairments and having insufficient knowledge of the Dutch language.

Participants were recruited from six Community Mental Health Teams (CMHTs) serving people with severe and persistent mental illness at Bavo Europoort, a mental health institution in the greater Rotterdam area in the Netherlands, a conurbation with approximately 1.2 million inhabitants. Each sub-region of about 150,000 inhabitants was served by a Community Mental Health Team and an Assertive Community Treatment Team (ACT).

Clients of the CMHTs who met the eligibility criteria were identified and asked by their clinicians to participate in IMR. From the start, most participants in IMR also participated in the pilot study.

The following activities were undertaken to prepare for the implementation of IMR in this institution: to visit Prof. Mueser and colleagues and to see IMR in practice, a study trip was made to Dartmouth Psychiatric Research Center New Hampshire, USA; an implementation plan was drawn up; plenary meetings were held with all outpatient clinicians at the institute and members of the clients council; a steering committee, an implementation committee and an education committee were established; translated handouts and workbooks were edited; a two-day training was held for 18 trainers and six supervisors; four supervision groups were established, a master class was given by Susan Gingerich, one of the creators of IMR; six IMR groups were started; and this pilot study was started.

### Interventions

In essence, IMR is a structured training consisting of 11 modules, practitioner guides and handouts for participants. The 11 modules are 1.) Recovery Strategies, 2.) Practical Facts about Mental Illness, 3.) the Stress-Vulnerability Model, 4.) Building Social Support, 5.) Using Medication Effectively, 6.) Drug and Alcohol Use and Treatment Strategies, 7.) Reducing Relapses, 8.) Coping with Stress, 9.) Coping with Problems and Persistent Symptoms, 10.) Getting Your Needs Met in the Mental health System, and 11.) Health for You.

The original American text had been translated into Dutch; where necessary, it was slightly adapted to the Dutch context. The IMR-training was given at the participating institute in a group format with weekly sessions. For the pilot, all six IMR groups completed the whole curriculum.

During the first module, which was the only module that was done individually, the participants decided which personal goals they wanted to work on during the program. For half of each 90-minute session, some participants worked on their goals in the group. During the other half of each session, all participants worked with the help of the handouts on the subjects of the modules.

Each IMR group was guided by two professional trainers (ten psychiatric nurses, one psychologist and one psychiatrist), who used 1.) motivation-enhancement strategies such as conveying confidence and exploring the pros and cons of change; 2.) educational strategies (psycho education) such as breaking down information, interactive teaching, and checking for understanding; and 3.) cognitive-behavioral techniques such as shaping, modeling and role-play. Peer-group support and coping & social skills training are integral to IMR. Homework assignments are provided.

Whether all different aspects of the intervention were actually administered is part of the fidelity, which was assessed for feasibility (see below).

### Objectives

#### Objectives and hypotheses of the main study

The aim of the RCT is to compare the effectiveness of the IMR training program with that of care as usual (CAU) for patients with SMI. Our hypotheses are inspired by Mueser’s conceptual framework [1, 10]. The first primary hypothesis of the main study is that IMR + CAU (where IMR is offered in group format) leads to better illness management and to fewer symptoms and relapses than CAU only. The second primary hypothesis is that, compared to CAU only, IMR + CAU leads not only to better “subjective” recovery (perceived recovery, sense of purpose, and personal agency), but also to better “objective” recovery (role and social functioning).

We also have four secondary hypotheses. 1.) We expect the cost-utility of IMR + CAU to be better than that of CAU. 2.) We expect better illness management (i.e., getting more insight, better coping, more social support, less addiction and better service engagement) to lead to fewer symptoms and relapses. Testing this will help us explore the working mechanisms of IMR. (3) The third hypothesis we will test is that, in terms of Mueser [1], better “distal outcomes” (i.e., recovery) result from a combination of better “proximal outcomes” (i.e., better illness management and fewer symptoms and relapses) and progress on personal goals. (4) Finally, we expect that any improvement resulting from IMR + CAU will be associated with the fidelity with which IMR is implemented.

#### Objectives of the pilot study

Our primary objectives concern the assessment of the feasibility of implementing the IMR training program on a broader scale at Bavo Europoort. As a satisfactory power level would be achieved only if we included enough patients in the planned RCT—we planned 200—we examined whether the institute would succeed in recruiting sufficient participants for the six IMR groups planned. The institution’s willingness to implement IMR in the long term would depend largely on clients’ and clinicians’ satisfaction with IMR, on which the effectiveness of the program would clearly have a bearing.

Our secondary objectives regard the assessment of the quality with which IMR is implemented. Because fidelity is considered to be associated with better outcomes [20–23], secondary objectives were to evaluate whether it was feasible to apply the 11 modules of IMR with satisfactory fidelity in the Dutch context and to set up an adequate infrastructure for trainers’ training and supervision, which is a precondition for implementing IMR with good fidelity. Further secondary objectives for the pilot study were to explore the duration of the program and dropout from it. The dropout percentage would give an indication of the number of completers of the intervention that could be expected in the main study. We also wanted to explore client characteristics related to dropout.

### Outcome measures for the main study

#### Illness management and illness outcomes

The primary outcome measure of the main study is the client version of the IMRS [10–12, 24]. One of the secondary outcome measures is the clinician-rated IMRS [10–12, 24]. The items of these two IMR scales mainly concern aspects of illness management and illness outcomes. Each of the two scales includes 15 items scored on a 5-point scale [11, 12]. Research on the IMR Scales indicates that internal consistency is moderate, that two-week test-retest reliability is high, and that the scales have convergent validity [24–28]. This supports the use of the IMR Scales in assessing illness management and recovery in people with severe mental illness.

#### Additional illness-management scales

Given the limited number of items in the IMR scales, the main study will also assess illness management using other validated and more comprehensive scales as secondary outcome variables, assessing coping, social support, treatment compliance, insight into illness, and problems with alcohol and drugs. To measure these topics, we will use the respective instruments: the Coping Self-Efficacy Scale (CSES) [29], the Multidimensional Scale of Perceived Social Support (MSPSS) [30], the Service Engagement Scale (SES) [31], the Insight Scale (IS) [32], and one item (item 24) of the Addiction Severity Index (ASI) [33, 34].

#### Illness outcomes: symptoms and relapses

The secondary outcomes on illness-management outcomes are symptoms, health complaints and functional limitations, and relapses. These topics will be measured with the Brief Symptom Inventory (BSI) [35–37] and the EQ-5D [38], with the number of relapses being operationalized as the number and duration of hospital admissions and the number of emergency-department visits.

#### Recovery

In Mueser’s conceptual framework [1], the concept of recovery is differentiated into subjective recovery and objective recovery. We will assess subjective recovery as secondary outcome variables using a generic personal recovery scale, and by using four measures to assess internal stigma, self-esteem and life-goals: The Mental Health Recovery Measure (MHRM) [39, 40] (authorized Dutch translation [41, 42]); the Internal Stigma of Mental Illness (Ismi) [43]; one item of the Quality of Life section of the Cumulative Needs for Care Monitor (CNCM) [44]; and the Self-Esteem Rating Scale-Short Form (SERS-SF) [45]. In line with Muesers’ conceptual framework, we view improving on personal goals as a mediator variable between illness self-management and recovery [1]. We will measure improving on personal goals with Granholm’s Goals Template [46]. Objective recovery will be assessed using the Social Functioning Scale [47].

#### Cost-utility

The number and duration of outpatient treatment contacts and inpatient days will be calculated on the basis of their cost in Euros, and are related with changes in quality of life measured by the EQ-5D [38]. Cost-utility can be calculated by transforming scores on the EQ-5D into quality-adjusted life-years (QALYs) [48].

#### IMR model fidelity

The researchers will determine model fidelity using the IMR Fidelity Scale [49], the IMR General Organizational Index (GOI) [2], and the Illness Management and Recovery Treatment Integrity Scale (IT-IS) [50].

### Outcomes

#### Outcome measures for the pilot study

Assessment of our primary objectives resulted in our primary feasibility outcomes, which regard 1) the number of participants, 2) clients’ and clinicians’ satisfaction with IMR, and 3) the effectiveness of the program. Assessment of our secondary objectives resulted in our secondary feasibility outcomes, which regard 1) fidelity, 2) setting up an infrastructure for education and supervision, 3) drop-out and completion, and 4) program duration. We also explored the completers’ characteristics.

Like program duration, participants’ recruitment, dropout and completion (i.e., participant numbers, participant characteristics related to dropout, and their reasons for dropout) are all measured by using monitoring data from the institute’s internal reports and registrations and by having interviews with clinicians. Completion of the program was defined as attendance of 70 % of the program sessions; non-completers were termed “dropouts from treatment”. Participants' and clinicians’ satisfaction with IMR were assessed in interviews with participants and clinicians. The infrastructure for trainers’ training and supervision was assessed in interviews with clinicians. The principal researcher (BJR) and one co-author (MB) carried out the semi-structured interviews. These two authors categorized and summarized data according to the topics of the interviews.

The fidelity of implementation of IMR per group was measured on the IMR Fidelity Scale [49]. The IMR Fidelity Scale is a scale to assess the degree of implementation of the IMR model. It consists of 13 items, each of which is rated on a five-point scale and each of which is behaviorally anchored; a score of five indicates full implementation. The other scale points represent an increasing degree of implementation [51]. The total score is the mean of all item-scores. Per group, fidelity measurement took almost a day, and was carried out by the principal researcher on the day of one of the last sessions. It consisted of semi-structured interviews with participants and the two trainers, plus observation of one session, and checking forms.

The effectiveness of IMR with regard to illness management and illness-management outcomes was measured with the Illness Management and Recovery Scale patient self-score version; and with the lllness Management and Recovery Scale clinician-rated version. Similarly, the effectiveness of IMR with regard to Recovery was measured with the Recovery Markers Questionnaire [RMQ] [52], a free-standing 24-item self-report subscale of the Recovery Enhancing Environment Measure (REE), which has a 5-point agreement-response scale ranging from “strongly agree” to “strongly disagree”. The scale has high internal and face validity [53, 54]. Measuring improvement on recovery is a secondary outcome measure of the main study.

### Sample size

For the pilot study we could include most people who intended to participate in the six IMR groups (*N* = 81). We did baseline measurements and gathered socio-demographic data on these participants.

### Feasibility criteria

Our primary feasibility criterion was whether the institute was able to recruit a sufficient number of participants for the six IMR groups planned for the pilot study. According to the IMR Fidelity Scale, the maximum number of participants in a group is eight [49]. We therefore needed 48 participants. Making allowance for some dropout, we thus aimed to recruit about ten people per group: 60 in total. We wanted to establish how many clients had to be asked to participate in IMR for this number to be reached. Our second primary feasibility criterion was the satisfaction of most participants and clinicians with IMR. The third was to achieve significant results on our effectiveness-related outcome measures.

Our first secondary feasibility criterion was that the institute could achieve total scores on the IMR Fidelity Scale of ≥ 4.0, which is considered to reflect good fidelity [51, 55]; in other earlier research on IMR, cut-off scores for good or high IMR fidelity were > 3.7 [6], > 3.8 [56]. In their review, McGuire et al. reported a weighted mean of 4.05 on fidelity for “all studies” (SD = .93) [5]. We also wished to identify the aspects of fidelity (as shown in item-scores) in which quality of implementation of IMR had to improve. Additionally, we tested whether the institute had successfully created a good infrastructure for trainers' supervision and training, our criteria for success being the institute's ability to fulfill its intention of completing a two-day training for all trainers, and of supervising trainers for two hours a week. The IMR review [5] refers to five studies in which training of trainers had taken two days [1, 51, 55, 57, 58]. While three other studies reported that training took 40 h [59], five days [8] and 48 h [6] respectively, the latter had involved only 50 % of the trainers.

On the assumption that dropout from treatment should be minimized, we wanted to establish how much dropout from treatment we could expect in the main study. We set no prior targets for the number of completers. The review of IMR studies refers to a median dropout rate from IMR of 24 % and a range of dropout rates from 18 % to 30 %; this review also refers to a median of 63 % completers and a weighted mean of 36 %, with a range of 15 %-86 % [5]. Of the four earlier RCTs, one reported an IMR drop-out rate of 21 % [6], and a second reported that 46 % of the participants assigned to the program attended fewer than half of the IMR sessions [7]. A third reported 5 % drop-out [8], but participants of this study had been selected “on the basis of consistent attendance of prior (non-IMR) services, and training and consultation focused heavily on consumer engagement” [5]. The fourth RCT reported that drop-out from IMR—defined as participating in less than half the scheduled groups—was 72 % [9]. Our fourth secondary feasibility criterion that would enable us to plan the main study properly is whether the duration of IMR was predictable. According to the review of IMR studies [5], 9-12 months is a usual length for a program consisting of one session per week; in three earlier RCT studies in which IMR was also applied in a weekly group format, IMR lasted for 8-11 months [6] and 9 months [8, 9].

### Statistical analysis

We used a paired sample *t*-test to measure the effectiveness of IMR (one group pre- and post- measurement) on the IMR-scale client version, the IMR-scale clinician version, and the RMQ. Chi square tests and independent samples t-tests were used to test differences between completers’ and non completers’ baseline characteristics.

### Ethical aspects

The study protocol, information brochure and informed consent form for the RCT were approved by the Dutch Union of Medical-Ethical Trial Committees for mental health organizations (registration number of the Dutch National Trial Register NTR 5033 http://www.trialregister.nl, CCMO-no NL38605.078.12).

The pilot study was a naturalistic study in which the researchers observed and recorded the implementation of IMR in the institute in its natural setting over a prolonged period, while interfering as little as possible with the subjects and the implementation process. Conducted by researchers employed at Bavo Europoort’s internal research and development department, it was implemented as a routine program evaluation under the regular quality control for care-innovation projects with a local scope. As its objective was not to gain generalizable medical scientific knowledge, it could not be defined as medical research according to the rules of the Dutch Central Committee on Research Involving Human Subjects (CCMO) [60] under the Dutch Medical Research Involving Human Subjects Act (WMO) [61]. For this reason, no external medical ethical permission was requested. The study was approved by the medical director of the institute.

Potential participants were provided with information on the program evaluation; participation in the evaluation was not a mandatory requirement for participation in the IMR program. Participants were asked individually whether they consented to be interviewed, and were asked their permission for the observation of one IMR-session per team for purposes of fidelity measurement. The outcomes were reported anonymously.

## Results

### Participant flow

#### Influx and outflux of participants in IMR

**Fig. 1:**
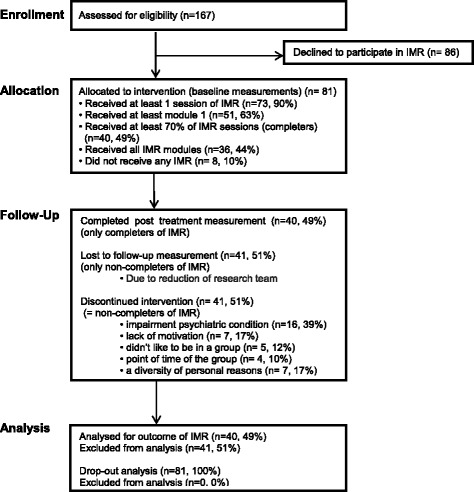
Flow Diagram Pilot study IMR

In the recruitment phase, 167 clients were asked by their clinicians to participate in IMR. Eighty-one who signed up to participate in IMR (49 %) were included in the study and had a first measurement; 73 of the 81 started with the first module (eight dropouts), which was done individually with one clinician. Fifty-one of these 73 participants started with the second module (22 dropouts), which, like the remaining modules, was given in group format. Forty of the 51 attended at least 70 % of all sessions (11 dropouts) and were thus considered to be completers (see Fig. 1). Dropout from the time of commitment to participate in IMR was thus 51 %; counted from the start of real participation, it was 45 %. Measured from the point at which clients had committed themselves to participating, dropout from treatment was highest between modules one and two, which coincided with the transition from the individual format to the group format. With regard to the 41 non-completers, their clinicians reported the following causes of dropout: worsening of psychiatric condition (16), lack of motivation (7), not liking being in a group (5); problems with the time at which the session was held (4), personal reasons such as moving away from the area, language problems, concentration problems or finding IMR too hard to do (7); and unknown (2).

After completing IMR, the participants were assessed for the second time. In a deviation from our original protocol, a sudden reduction in the research team meant we could not conduct follow-up measurements of the non-completers.

### Recruitment

The pilot study started at the beginning of 2009. The period for recruiting participants and for baseline measurements lasted from April 2009 to January 2010. The period of second measurements lasted from April 2010 to December 2010. Data collection took place in 2009, 2010 and 2011. Data-analysis took place in 2012, 2014 and 2015.

### Baseline data

#### Participants’ characteristics

Forty-eight participants in the pilot study (*N* = 81) were men (59 %). On average, participants were aged 42. Forty percent lived alone, 39 % had attended only lower education, 9 % had income from employment, 28 % had not been born in the Netherlands, 54 % had psychiatric problems that had started over 10 years before, and 56 % had been hospitalized at least once. Seventy-three percent had been diagnosed as having a psychotic disorder, 54 % had a length of treatment of more than five years, and 20 % had previously been hospitalized for longer than one year, see Table 1.

**Table 1:** Characteristics at baseline of completers of IMR versus non- completers

		Total *N* = 81			Completers *N* = 40		Dropouts from IMR *N* = 41		χ^2^ (*df* = 1)	*p*	*OR*	95 % CI
gender	male	48	59 %		19		29		4.53	.033*	2.67	[1.07, 6.67]
	female	33	41 %		21		12					
living situation												
living alone		32	40 %		19		13		2.18	.139	0.48	[0.18, 1.28]
living with others		34	42 %		14		20					
missing		15	18 %									
education level												
low		31	39 %		14		17		0.81	.369	1.59	[0.58, 4.36]
middle + high		30	37 %		17		13					
missing		10	24 %									
native country												
born in Holland		42	52 %		22		20		0.47	.492	0.70	[0.25, 1.95]
immigrant		23	28 %		10		13					
missing		16	20 %									
diagnosis												
psychotic disorders		59	73 %		31		28		0.58	.446	0.68	[0.25, 1.85]
All other diagnoses		21	26 %		9		12					
missing		1	1 %									
start of problems												
<10 years ago		20	25 %		8		12		0.55	.457	1.50	[0.51, 4.38]
>10 years ago		44	54 %		22		22					
missing		17	21 %									
length of treatment												
<5 years		17	21 %		6		11		1.42	.234	2.01	[0.63, 6.39]
>5 years		44	54 %		23		21					
missing		20	25 %									
number of admissions												
None		12	15 %		6		6		0.04	.837	0.88	[0.25, 3.13]
≥1		45	56 %		21		24					
missing		24	29 %									
length hospitalization												
<1 year		37	46 %		18		19		0.01	.928	1.06	[0.33, 3.41]
>1 year		16	20 %		8		8					
missing		28	35 %									
source of income												
employment, unemployment or invalidity benefit		46	57 %		27		19		4.26	.039*	0.29	[0.09, 0.97]
social security benefit		17	21 %		5		12					
missing		18	22 %									
				*M (SD)*	*N*	*M (SD)*	*N*	*M (SD)*	*t (df)*		Cohen’s *d*	
Age		81	100 %	42.35 (10.12)	40	44.03 (8.70)	41	40.71 (11.20)	1.49 (75)	.140	0.33	[0.11, 0.77]
IMR-scale client version at baseline		66	82 %	3.36 (0.42)	36	3.46 (0.39)	30	3.24 (0.42)	2.21 (64)	.031*	0.55	[1.04, 0.05]
IMR-scale clinician version at baseline		70	86 %	3.09 (0.50)	37	3.22 (0.44)	33	2.94 (0.53)	2,41 (68)	.019*	0.58	[1.06, 0.10]
Recovery Markers Questionnaire [RMQ]		62	77 %	12.4 (5.76)	33	12.5 (5.55)	29	12.3 (6.1)	0.14 (60)	.890	0.04	[0.54, 0.46]
				*Mdn/IQR*		*Mdn/IQR*		*Mdn/IQR*	*U*	*p*	*r*	
GAF		79	98 %	50/10	39	55/10	40	50/10	591	.058	-.21	[-0.42, 0.01]

*significant *p* < .05

### Outcomes and estimation

Table 2 gives an overview of the feasibility objectives, outcome measures, feasibility criteria and outcomes of the IMR pilot study.

**Table 2:** Overview of IMR pilot study (main feasibility objectives and outcomes)

	Objectives	Outcome measures	Feasibility criteria	Outcomes
1	To include sufficient participants	Monitoring; interviews	60 people	81 people included of 167, assessed for eligibility (49 %); sufficient for 6 IMR groups
2	Clients’ and clinicians’ satisfaction with IMR	Interviews	Satisfaction of most participants and clinicians with IMR	Completers and trainers:(very) positive about IMR
3	IMR outcome (pre-post design)	IMR Scale client version (Mueser et al. 2004) IMR Scale clinician version (Mueser et al. 2004) RMQ (Ridgway et al. 2003)	Significant results on our outcome measures	IMRS clinician version (*p* < 0.001, *d* = 0.84)^a^ IMRS client version (*p* = 0.063; *d* = 0.41) RMQ (*p* = 0.003, *d* = 0.52)^a^.
4	Satisfactory fidelity	IMR fidelity scale (Mueser et al. 2009)	Total scores on the fidelity scale of at least 4.0.	Total score (six groups): Mean (SD) = 4.0 (0.20) Three groups: total scores ≥ 4.0 Three groups: total scores < 4.0
5	Trainers' education and supervision	Interviews	Two-day training and Supervision of two hours per week	Two-day course before start of the pilot; Supervision bi-weekly for two hours; Additional training 2 x per year for 4 h.
6	Dropout from IMR	Monitoring; interviews	Exploration, no targets set	Dropout from IMR of 51 %
7	Duration of the IMR-program	Monitoring; interviews	Predictable; 9–12 months	M = 12.7 months, SD = 3.14

^a^Significant improvement in completers

#### A. Primary feasibility outcomes

##### Number of participants

The institute succeeded in running the six IMR groups planned, all of which completed the whole curriculum and which, between them, succeeded in recruiting 81 participants of 167 people assessed for eligibility, so more than the 60 participants needed; see “influx and outflux of participants in IMR,” above.

##### Satisfaction

On the day of the last session of their IMR group, or some days later, 20 completers were interviewed (50 % of all completers), 17 in group interviews and three individually. All twelve clinicians who had trained the six IMR groups were interviewed individually.

Completers were very positive about IMR. Most of all, they wanted to achieve the following: to obtain knowledge (13 respondents); to increase insight (13 respondents); the contact with fellow patients (peer-contact) (9 respondents); to work on personal goals (8 respondents); and to learn coping skills (also named 8x).

All IMR-trainers were positive about IMR; some said that they saw changes in individual clients. They appreciated the content of the training, adding, for example, that it was very structured *and “nice and fun to give.”* Trainers appreciated the recovery vision of IMR. Some thought that participants learn most by practicing social skills such as assertiveness. They appreciate it if there is a lot of interaction in the group and if role-play and exercises can be done according to the topics of the modules. They thought that peer contact helps and that participants are motivated to meet each other. Some trainers observed that participants adopted coping mechanisms from each other. Most trainers said that, as safety in the group is an important condition for people to open up, they preferred to have a “closed group,” i.e., no rolling admissions.

##### Effectiveness

Completers showed a significant improvement on the IMR Scale clinician version (*N* = 36); and also on the RMQ (*N* = 31). Completers did not show a significant improvement on the IMR Scale client version (*N* = 33), see Table 3.

**Table 3:** Effectiveness of IMR completers (one group pre- and post- measurement; paired sample *t*-test)

	*N*	*M (SD) pre*	*M (SD) post*	*t*	*Df*	*p*	*d*	95 % CI
IMR-scale client version	33	3.47 (0.39)	3.66 (0.50)	1.93	32	.06	0.41	[-0.08, 0.90]
IMR-scale clinician version	36	3.21 (0.44)	3.59 (0.48)	4.73	35	<.001	0.84	[0.36, 1.32]
RMQ	31	12.23 (5.61)	14.94 (4.84)	3.19	30	.003	0.52	[0.01, 1.02]

#### B. Secondary feasibility outcomes

##### Fidelity

The mean (SD) total fidelity score of the six groups was 4.0 (.20), which meets the stated objective of ≥ 4.0. The median (IQR) was 3.9 (.35). Three groups had overall good fidelity, with total scores on the IMR Fidelity Scale [49] of 4.0, 4.2 and 4.2. But three groups had moderate fidelity, all with total scores of 3.8. Eight items with scores ranging from 4.2 to 5.0 reflected good implementation: “Number of People in a Session or Group,” “Program Length,” “Comprehensiveness of the Curriculum,” “Provision of Educational Handouts,” “IMR Goal Setting,” “Motivation-Based Strategies,” “Educational Techniques” and “Cognitive-Behavioral Techniques”. Four aspects were implemented poorly in all groups: “Involvement of Significant Others,” “Coping-Skills Training,” “Relapse-Prevention Training” and “Behavioral Tailoring for Medication.” The greatest variance was on item 7 regarding “IMR Goal Follow-up,” which was implemented well in 3 groups and not implemented well in 3 groups; the items score ranged from 1–5, see Table 4.

**Table 4:** Fidelity-scores on the IMR-fidelity scale of the six IMR groups

	Group	A	B	C	D	E	F	*M (SD)*	Mdn (IQR)
	Item								
1	# of people in a Session or Group	4	5	5	5	4	5	4.7 (.52)	5 (.75)
2	Program Length	5	5	5	5	5	5	5 (0)	5 (0)
3	Comprehensiveness of the Curriculum	5	5	5	5	5	5	5 (0)	5 (0)
4	Provision of Educational Handouts	5	5	5	5	5	5	5 (0)	5 (0)
5	Involvement of Significant Others	3	1	3	3	3	3	2.7 (.82)	3 (0)
6	IMR Goal Setting	5	5	5	5	5	5	5 (0)	5 (0)
7	IMR Goal Follow-up	5	4	2	5	1	2	3.2 (1.72)	3 (2.75)
8	Motivation-Based Strategies	5	4	4	5	5	5	4.7 (.52)	5 (.75)
9	Educational Techniques	5	5	5	5	5	5	5 (0)	5 (0)
10	Cognitive-Behavioral Techniques	5	4	4	5	4	4	4.3 (.52)	4 (.75)
11	Coping Skills Training	3	2	2	4	3	2	2.7 (.82)	2.5 (1)
12	Relapse-Prevention Training	2	2	3	1	3	3	2.3 (.82)	2.5 (1)
13	Behavioral Tailoring for Medication	2	2	2	2	1	3	2 (.63)	2 (0)
	Mean fidelity score per group	4.2	3.8	3.8	4.2	3.8	4.0	4.0 (.20)	3.9 (.35)
	Median fidelity score (IQR)	5 (2)	4 (3)	4 (2)	5 (1)	4 (2)	5 (2)		

Due to a ceiling effect, the differences between groups perceived for some items in the quality of implementation during observation of one session per team were reflected only partly in the scores of the fidelity scale. In particular, this concerned items 8 (motivation-based strategies), 9 (educational techniques), and 10 (cognitive-behavioral techniques). Variance in fidelity appeared to be related to differences in the trainers’ skills and their experience in guiding groups. This was reflected particularly in their ability to practice social and coping-skills training, role-play, relapse-prevention training, and motivational strategies.

##### Infrastructure for education and supervision

By setting up a system for the supervision and training of the trainers, the institute successfully created an infrastructure for implementing IMR on a broader scale. The trainers were experienced clinicians (mostly psychiatric nurses, but also a psychologist and a psychiatrist), who 1.) all received a two-day course in teaching IMR before the start of the pilot and 2.) attended supervision once every two weeks for two hours. Twice a year since the start of the pilot, all IMR trainers have come together for a morning or afternoon of additional booster training.

##### Dropout and completion

Measured from the point at which clients committed themselves to participation, dropout from IMR was 51 %; measured from the moment of actual participation, it was 45 %. The number of participants per IMR group at the start ranged from six to twelve, and the number in the last sessions from three to nine. Four groups were “closed groups” (no new influx at dropout); two groups had “rolling admissions”.

Defined as > 70 % participation, completion of IMR was 49 %; measured from the moment of actual participation, it was 55 %. If program completion is defined as having received all IMR modules [5], the completion rate was 44 %; measured from the moment of actual participation, it was 49 % (see Participant Flow, above, and Fig. 1).

##### Duration

While the average length required per module lay between three and four 90-min sessions, it could vary between groups between one and eight sessions, according to the module and the group’s preference or specific challenges. The durations of the IMR training for the six groups were 8, 11, 12, 13, 15 and 17 months (*M* = 12.7 months, *SD* = 2.87).

##### Completers’ characteristics at baseline

Significantly more women than men completed IMR, and, at baseline, completers scored significantly better than non-completers on the client and clinician versions of the IMR scale.

An association between completion and source of income showed that those in employment, those receiving unemployment benefit and those receiving invalidity benefit had a significantly greater chance of completing IMR than those receiving social security benefit. To us, this suggests that people who dropped out were at a greater distance from everyday working life, and possibly have more problems in maintaining structured and social activities than completers. There were no significant differences between completers and non-completers on the baseline scores of the Recovery Markers Questionnaire (RMQ) or the Global Assessment of Functioning scale (GAF). Neither were there associations between completion with living situation, education level, native country, diagnosis, period of start of problems, length of treatment, number of admissions, and length of hospitalization, see Table 1.

## Discussion

### Interpretation

As the primary objectives of this pilot study concerned the feasibility of implementing the IMR training program on a broader scale, we measured the following: 1.) whether the institute had succeeded in recruiting sufficient participants for the planned six IMR groups, 2.) clients’ and clinicians’ satisfaction with IMR, and 3.) the effectiveness of the program.

That the institute could recruit 81 IMR participants to the pilot study from the 167 people assessed for eligibility means that we exceeded our feasibility criterion of 60. This suggests that a good number of clients is interested in this treatment. It also supports the feasibility of getting sufficient participants for the RCT, although recruiting for an RCT is probably harder than recruiting for a pilot study with no control group.

The satisfaction of the completers interviewed and of all clinicians was very good. However, as we measured this only at follow-up, we could not measure change over time, and as we did not interview people who dropped out, we know only that 49 % of all participants were satisfied with IMR.

IMR was effective for completers on the IMR-scale clinician version, with a large effect-size *d* = 0.84, and on the RMQ, with a medium effect size (*d* = 0.52), but there was no significant improvement on the IMR-scale client version (*d* = 0.41). However, a limitation of this pilot study is our deviation from the original protocol: due to a sudden reduction in the research team, we could not conduct follow-up measurements or interview the non-completers; neither did we have a control group. We nevertheless conclude that our institute appears to have a sufficiently firm support base for implementing IMR on a broader scale, and that this contributes to the feasibility of an RCT.

The secondary objectives of the pilot study were to implement IMR with satisfactory fidelity, to create a sufficient infrastructure for the trainers’ education and supervision, to explore program duration, and to explore dropout.

With regard to the RCT, we see it as an advantage that, due probably to sufficient trainer education and supervision, the institute successfully established six IMR groups in this pilot period whose average total fidelity score on the IMR Fidelity Scale was 4.0. Therefore our feasibility criterion of ≥ 4.0 was met. We also see it as very useful that our item scores helped us identify the aspects of implementation of IMR that the institute must improve to achieve total scores ≥ 4.0 for all groups. Because the results of the interviews suggested that variance in fidelity is related to the differences in the trainers’ professional skills, this gives input for further education and supervision. We have decided to add the IT-IS scale [50] to the RCT, as it focuses on measuring clinicians’ skills related to fidelity.

One of our feasibility criteria for a good infrastructure for education and supervision was to give all 12 trainers two days’ training, and to supervise them for two hours per week. A two days’ training was indeed achieved for all 12 trainers, and the trainers had eight hours of supplemental training in one year. These 24 h were more than the length of training in five studies in McGuire’s review, but less than in three other studies (one of which had involved only 50 % of the trainers) (see above). Although the institute had intended to provide supervision once weekly, productivity requirements meant that trainers could attend supervision only once every two weeks for two hours throughout the duration of the pilot study and thereafter. This was more intensive than in one RCT, which reported monthly supervision [6], but less intensive than in another, which reported weekly supervision [8]. Because, in one year, the institute successfully achieved a good average total fidelity score of 4.0, we suggest that fidelity may be further improved by continuing such education and supervision, especially if the focus lies on aspects of fidelity that most need improvement (see above). Although our results on dropout from IMR were worse than those reported in the review on IMR [5], and also than those in one earlier RCT on IMR [6], they do not appear to be worse than the numbers in two other RCTs [7, 9], even though the overall results are not entirely comparable: in our study, dropout is defined as < 70 % participation. But with regard to the planned RCT, we conclude that if no measures are taken to reduce it, allowance should be made for substantial dropout from IMR.

McGuire et al. [5] report a median completion rate of 63 % with a range of 15 %-86 %. If we adopt his definition of program completion—having received all IMR modules, which in our study would mean a completion rate of 44 %— our result falls well within this range.

But as McGuire et al. said with regard to the studies they reviewed, we feel that the dropout and completion rates found in our study “leave much room for improvement.” For the RCT, we plan to recommend clinicians and managers to pay attention to this aspect and to take various measures including the use of a good dropout protocol. Our baseline analysis of completers’ and non-completers’ characteristics also suggest that, to reduce dropout from IMR, special attention is required by male participants, people who receive social security benefit, and people who score lower on the IMR scales at baseline. And although “intention to treat” will be used in the RCT, we have decided that a ratio of 3:2 will be used for randomization. This will enable us to include more participants in the experimental condition, and will provide enough power for secondary analysis of effectiveness for completers.

The different durations of the IMR-training—which ranged from 8 to 17 months (*M* = 12.7 months, *SD* = 2.87)—were on average somewhat longer than the 9 to 12-month range mentioned in McGuire’s review, and also longer than those in three earlier RCT studies in which IMR was applied in a weekly group format [6, 8, 9]. The durations of 15 and 17 months of two groups are a particular cause for concern. For properly feasible planning of the RCT, we have therefore asked the institute to try to maximize IMR length at around 12 months. This is because if we plan the first follow-up measurement after one year in the RCT, an IMR length of longer than 1 year would mean that participants would not have completed the whole curriculum, which might be seen as a disadvantage.

## Conclusions

The main objective of this pilot study was to assess the feasibility of conducting an RCT. Our results with regard to the recruitment of sufficient participants, to clients’ and clinicians’ satisfaction with IMR, and to the effectiveness of the program all suggest the feasibility of our primary objectives for this pilot study, which regard implementing the IMR-training program on a broader scale at Bavo Europoort.

The feasibility of an RCT is also suggested by the results with regard to our secondary objectives regarding the quality of implementation: to implement IMR with satisfactory fidelity, to set up a proper infrastructure for education and supervision, and to achieve an acceptable dropout percentage and a predictable program duration. If the institute is willing to follow our recommendations on recruitment, improving fidelity, training and supervision, and duration of the program, feasibility might even be greater.

## Generalizability

A precondition for generalizability of the feasibility found in this pilot study is that other institutions have the same drive to create a comparable infrastructure for implementing and sustaining IMR, and also have comparable potential for doing so: this is because implementing six IMR groups from scratch required a considerable effort on the part of the institute.

This pilot study has limitations that impair generalizibility of some results. As stated above: it was only for completers—49 % of all participants—that we could measure effectiveness and satisfaction. As the RCT was planned for implementation in the same institution, and as the pilot study was a naturalistic study, interfering as little as possible with the subjects and the implementation process, good generalizability of the other results of the pilot study to the RCT was probably to be expected. But although recruitment in the IMR pilot went well, we suppose that recruiting for an RCT will be harder.

## Abbreviations

ASI: Addiction Severity Index
BSI: The Brief Symptom Inventory
CAU: Care as usual
CCMO: Centrale Commissie Mensgebonden Onderzoek (Central Committee on Research involving Human Subjects)
CNCM: the Cumulative Needs for Care Monitor
CSES: The coping self-efficacy scale
EQ-5D: Euro-Qol 5D
GAF: Global Assessment of Functioning
GOI: General Organizational Index
IMR: Illness management and recovery
IMRS: Illness management and recovery scale
IQR: Interquartile range
IS: The Insight Scale
ISMI: The Internal Stigma of Mental Illness
IT-IS: Illness Management and Recovery Treatment Integrity Scale
MHRM: The Mental Health Recovery Measure
MSPSS: The Multidimensional Scale of Perceived Social Support
QUALYs: Quality adjusted life years
RCT: Randomized controlled trial
RMQ: Recovery Markers Questionnaire
REE: Recovery Enhancing Environment Measure
SAMHSA: Substance Abuse and Mental Health Services Administration
SERS-SF: The Self-Esteem Rating Scale-Short Form
SES: The Service Engagement Scale
SMI: Serious mental illness
WMO: Wet Medischwetenschappelijk Onderzoek met mensen (Medical Research Involving Human Subjects Act)
